# Role of BCL9L in transforming growth factor-β (TGF-β)-induced epithelial-to-mesenchymal-transition (EMT) and metastasis of pancreatic cancer

**DOI:** 10.18632/oncotarget.12455

**Published:** 2016-10-04

**Authors:** Giuseppina Sannino, Nicole Armbruster, Mona Bodenhöfer, Ursula Haerle, Diana Behrens, Malte Buchholz, Ulrich Rothbauer, Bence Sipos, Christian Schmees

**Affiliations:** ^1^ Natural and Medical Sciences Institute (NMI) at the University of Tuebingen, Tumor Biology Group, Reutlingen, Germany; ^2^ Experimental Pharmacology and Oncology GmbH, Berlin, Germany; ^3^ Department of Medicine, Division of Gastroenterology, Endocrinology and Metabolism, Philipps University Marburg, Marburg, Germany; ^4^ Pharmaceutical Biotechnology, University of Tuebingen, Tuebingen, Germany; ^5^ Institute of Pathology, University of Tuebingen, Tuebingen, Germany; ^6^ Current address: Institute of Pathology, Laboratory of Pediatric Sarcoma Biology, Ludwig-Maximilians-Universität Munich, Munich, Germany; ^7^ Current address: Department of Internal Medicine II, University of Tuebingen, Tuebingen, Germany

**Keywords:** pancreatic cancer, BCL9L, β-catenin, E-cadherin, EMT

## Abstract

Pancreatic ductal adenocarcinoma (PDAC) has a low overall survival rate, which is approximately 20% during the first year and decreases to less than 6% within five years of the disease. This is due to premature dissemination accompanied by a lack of disease-specific symptoms during the initial stages. Additionally, to date there are no biomarkers for an early prognosis available.

A growing number of studies indicate that epithelial to mesenchymal transition (EMT), triggered by WNT-, TGF-β- and other signaling pathways is crucial for the initiation of the metastatic process in PDAC. Here we show, that BCL9L is up-regulated in PDAC cell lines and patient tissue compared to non-cancer controls. RNAi-induced BCL9L knockdown negatively affected proliferation, migration and invasion of pancreatic cancer cells. On a molecular basis, BCL9L depletion provoked an increment of E-cadherin protein levels, with concomitant increase of β-catenin retention at the plasma membrane. This is linked to the induction of a strong epithelial phenotype in pancreatic cancer cells upon BCL9L knockdown even in the presence of the EMT-inducer TGF-β. Finally, xenograft mouse models of pancreatic cancer revealed a highly significant reduction in the number of liver metastases upon BCL9L knockdown. Taken together, our findings underline the key importance of BCL9L for EMT and thus progression and metastasis of pancreatic cancer cells. Direct targeting of this protein might be a valuable approach to effectively antagonize invasion and metastasis of PDAC.

## INTRODUCTION

Pancreatic ductal adenocarcinoma (PDAC), the most frequent type of pancreatic cancer [1, 2], has a low overall survival rate of ~ 20% during the first year after diagnosis and less than 6% within five years [3]. In 2015, the deceases for pancreatic cancers are predicted to raise for both men and women to a total number of 85,300 [4]. Currently, the only curative treatment available for PDAC is surgical resection, which, however, is suitable mainly for disease stages I and II, representing 10–15% of all patients. The remaining majority, mostly at stage IV, presents high rates of local recurrence due to resistance to radiochemotherapy [5, 6]. The low overall survival rate of this cancer is due to its premature dissemination [7] accompanied by a lack of disease-specific symptoms and reliable biomarkers detectable in the initial stages [8]. Recently, it has been shown that pancreatic cancer cells spread before and in parallel to establishment of the primary tumor, providing a potential explanation to the presence of metastasis in the majority of patients with pancreatic cancer already at the time of diagnosis [7]. Thus, a detailed understanding of the metastatic process of pancreatic cancer and the regulatory molecules involved is fundamental to improve its survival rate and develop more efficient therapies.

A growing number of studies support the notion that epithelial to mesenchymal transition (EMT) is crucial for the initiation of the metastatic process. Key to EMT is the acquisition of a mesenchymal phenotype by epithelial tumor cells, accompanied by a loss of epithelial marker expression (incl. E-cadherin), disruption of cell adherens junctions, and the gain of spindle-like shape and increased ability to migrate and invade, finally resulting in the generation of a secondary tumor [9]. Additionally, WNT signaling is known to synergize with other pathways, such as TGF-β, to trigger the EMT process [10–12]. WNT target genes, as for example SNAIL- and ZEB-family members, have been shown to repress E-cadherin expression leading to the loss of the epithelial in favor of a mesenchymal phenotype [13, 14].

The WNT pathway is known to be essential for pancreas development [15]. Global genomic analyses revealed that, in the prevalence of PDAC cases, the WNT pathway is one of the most strongly altered signaling pathways [16, 17]. In the absence of WNT ligand, β-catenin, the central component of canonical WNT signaling, is located at the plasma membrane, where it interacts with E-cadherin and α-catenin ensuring cell-cell adhesion [18, 19]. Activation of the WNT pathway causes the inhibition of the β-catenin destruction complex followed by cytoplasmic enrichment and translocation of β-catenin to the nucleus. Here, β-catenin binds to a variety of co-factors including BCL9/BCL9L and Pygopus proteins. Finally, this protein complex interacts with transcription factors of the LEF/TCF family triggering the transcription of WNT target genes [20]. BCL9 (B-cell CLL/lymphoma 9) and its homologue BCL9L (B-cell CLL/lymphoma 9-like, BCL9-2, DLNB11) have previously been shown to be associated with the formation of leukemia and other human malignancies [21]. To date, the role of BCL9L in malignancy has been studied mainly in colon cancer, where it was found to be a β-catenin interaction partner and to enhance its TCF-mediated transcription [22, 23]. Further studies implicated a role for BCL9L in the initiation of colon cancer as well as its involvement in the EMT process of this tumor entity [24, 25]. In a recent study, a role for BCL9L during induction of ER-positive breast cancer has also been described [26].

Using *in vitro* as well as *in vivo* model systems we demonstrate the importance of BCL9L for the progression of pancreatic cancer and propose a novel, so far unknown functional role of BCL9L in the regulation of EMT. Quantification of mRNA expression levels shows that BCL9L expression is significantly up-regulated in patient-derived PDAC tissues compared to tissues derived from non-cancer and chronic pancreatitis patients. RNAi mediated knockdown studies revealed an impairment of cell proliferation, migration and invasion of pancreatic cancer cells. On a molecular level, we found that BCL9L depletion provokes an increment of E-cadherin protein levels, with concomitant increase of β-catenin retention at the plasma membrane. We demonstrated that the BCL9L specific knockdown induces a strong epithelial phenotype in pancreatic cancer cells even after treatment with the EMT-inducer TGF-β. Results obtained from xenograft mouse models of pancreatic cancer confirmed the relevance of BCL9L for tumor growth *in vivo* and showed a highly significant reduction in the number of liver metastases upon BCL9L knockdown. Taken together, our findings underline the key importance of BCL9L for EMT and thus progression and metastasis of pancreatic cancer cells.

## RESULTS

### BCL9L is up-regulated in pancreatic cancer tissue and cell lines

**Figure 1:**
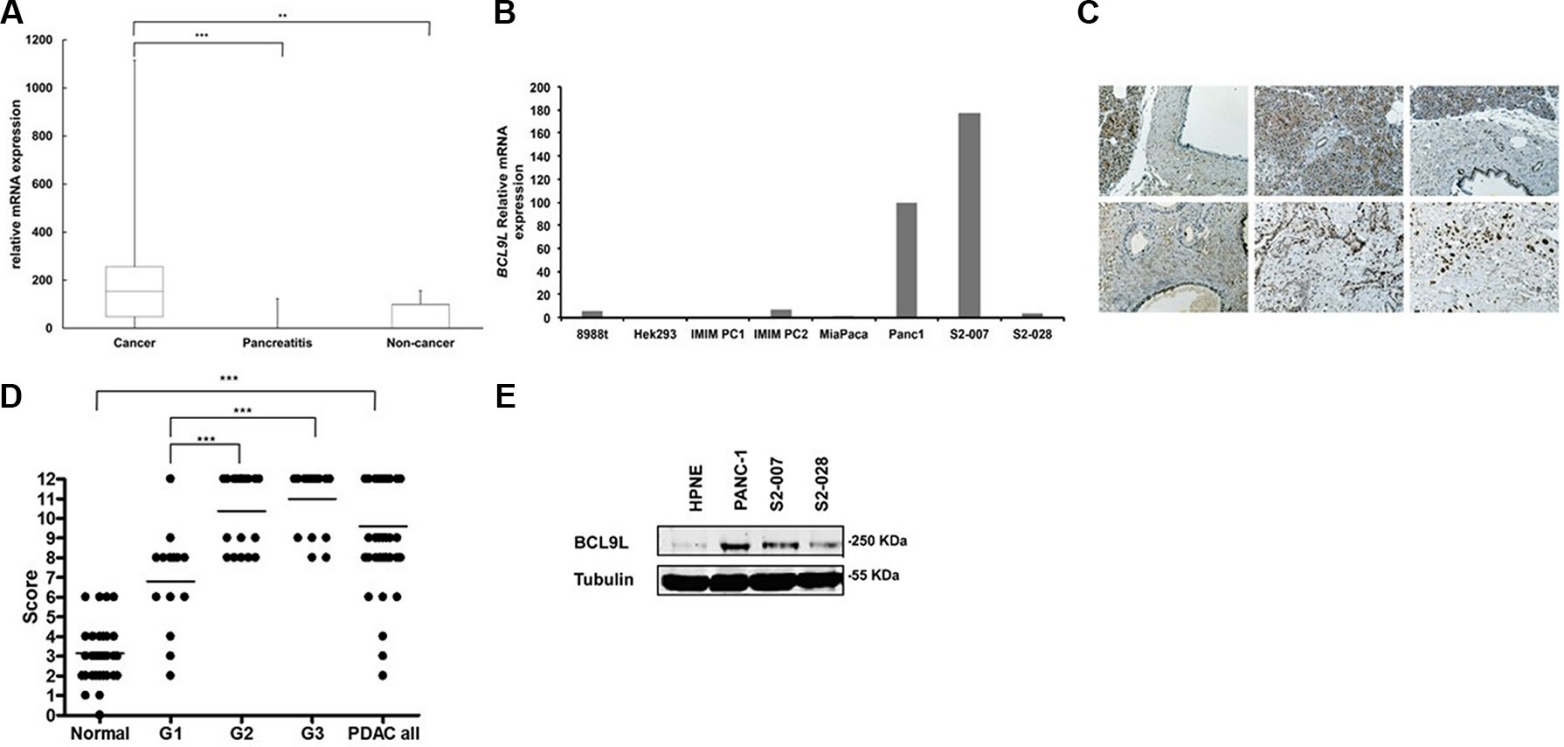
BCL9L expression in primary pancreatic tumor tissue and cell lines (**A**) Box-and-whisker plot showing results from BCL9L mRNA expression analysis by qRT-PCR in tissue samples derived from primary human pancreatic tumors (*n* = 26 cases), chronic pancreatitis (*n* = 6 cases) and normal pancreas (*n* = 13 cases). Expression was normalized to ribosomal protein, large, P0 (RPLP0) mRNA levels. Bars represent median and 2nd and 3rd quartiles (boxes) as well as minimum and maximum values (whiskers). **p ≤ 0.01, ***p ≤ 0.001 (Student's *t-test*). (**B**) BCL9L mRNA levels in pancreatic cancer (8988t, IMIM PC1, IMIM PC2, MiaPaca-2, Panc1, S2-007, S2-028) and control (HEK 293) cell lines. (**C**) Staining of tissue microarrays (TMA) for BCL9L expression using immunohistochemistry. Enhanced expression was seen in PDAC tissues (bottom panels) compared to normal pancreas (upper panels). Staining intensity in PDAC tissues increased with tumor grade (bottom left: G1, bottom middle: G2, bottom right: G3). (**D**) BCL9L staining intensity of tissue microarrays was quantitatively evaluated and scored in normal and PDAC cases as described in Materials and Methods. Scores significantly increased with the progression to less differentiated PDAC (grade 2 and 3). ***p ≤ 0.001 (Mann-Whitney and Kruskal-Wallis non parametric test) (**E**) BCL9L protein expression in pancreatic cancer and control (HPNE) cell lines was quantified by western blotting. Detection of α-tubulin was used as a loading control. Shown is a representative image of 3 experiments.

Levels of BCL9L mRNA were determined in tissues from patients with primary pancreatic cancer and chronic pancreatitis using qRT-PCR and compared with expression levels in pancreas tissue from healthy individuals. In total 26 cancer, six chronic pancreatitis and 13 healthy pancreas tissue samples were analyzed. BCL9L gene expression was detected in 80% of PDAC cases and significantly elevated compared to chronic pancreatitis and healthy pancreas tissues (Figure 1A). Additionally, we analyzed BCL9L mRNA (Figure 1B) expression in HEK293 cells as well as seven pancreatic cancer cell lines including Panc-1 and MiaPaca-2 [27], derived from pancreatic primary tumor tissue, and S2-007 and S2-028 representing sub-lines of SUIT2, a human pancreatic tumor cell line derived from liver metastasis tissue. In this context, S2-007 has been characterized as a moderately differentiated and highly metastatic tubular adenocarcinoma and S2-028 was shown to be a papillo-tubular adenocarcinoma and rarely metastatic [28]. Compared to S2-028 and MiaPaca-2 cells we determined increased BCL9L protein and mRNA levels in Panc-1 and S2-007 cells (Figure 1B). These findings were further validated by analysis of BCL9L protein levels in primary human tissues and cultured cell lines. Immunohistochemical stains revealed a nuclear reaction with anti-BCL9L antibody in normal ducts, in acinar cells and virtually all PDACs (Figure 1C). Acini and normal ducts were mostly weakly or moderately stained (mean score for ducts 3.16, SD: 1.54). PDAC exhibited significantly higher BCL9L expression (mean score 9.6, SD: 2.62) than normal duct cells (Mann—Whitney *U* test, *p* < 0.001; Figure 1D). Less differentiated PDAC (Grade 2 and 3) showed very strong BCL9L staining (mean scores 10.4 (sd 1.83) and 11.0 (sd 1.55), respectively), in contrast to moderate expression of BCL9L in well differentiated tumors (6.8, sd 2.3). This difference was also significant (Kruskal-Wallis, *p* < 0.001; Figure 1D). In congruence, increased BCL9L protein levels were detected in pancreatic cancer cell lines used for subsequent functional experiments vs a normal human pancreatic cell line (HPNE) (Figure 1E).

These findings strongly suggest a correlation of BCL9L expression with pancreatic cancer formation.

### BCL9L regulates proliferation, migration and invasion of pancreatic cancer cells

**Figure 2:**
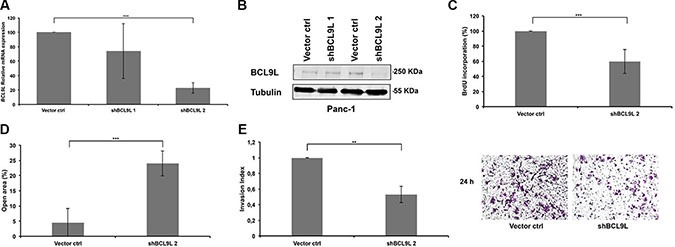
RNAi-mediated knockdown of BCL9L diminishes proliferation, migration and invasion of pancreatic cancer cells Relative levels of BCL9L mRNA (**A**) and protein expression (**B**) were determined in Panc-1 cells stably transduced with BCL9L-shRNA and control constructs by qRT-PCR and western blotting, respectively. shRNA-knockdown using the shBCL9L_2 construct resulted in ~80% reduction of BCL9L mRNA expression and almost complete abrogation of BCL9L protein expression compared to control cells. BCL9L mRNA levels were normalized to GAPDH. Tubulin served as the loading control in western blotting experiments. Shown is a representative image of 3 experiments (**C**) Stable knockdown of BCL9L significantly reduced the proliferative capacity of Panc-1 cells as quantified by DNA BrdU incorporation assay. (**D**) Wound healing assays revealed significantly impaired migration of Panc-1 cells stably transduced with BCL9L shRNA compared to controls. (**E**) Analysis of cell invasion showed a significantly reduced ability of BCL9L-knockdown Panc-1 cells to invade through a matrigel-coated transwell membrane in comparison to control cells. Right panel shows representative phase-contrast images of crystal-violet-stained BCL9L-knockdown (right) and control cells (left) on the lower side of a transwell membrane. All data represent mean ± s.e.m. of at least three independent experiments. **p ≤ 0.01, ***p ≤ 0.001 (Student's *t-test*).

In order to study the functional relevance of BCL9L up-regulation in pancreatic cancer, its expression was stably silenced in Panc-1 cells using two different shRNAs targeting the coding sequence (CDS; shBCL9L_1), and the 3′UTR (shBCL9L_2), respectively. Expression of both shRNA constructs led to down-regulation of BCL9L on mRNA levels (Figure 2A). As shBCL9L_2 also substantially diminished BCL9L protein levels (Figure 2A–2B), subsequent experiments were performed with cells stably expressing shBCL9L_2 in parallel to transient transfection experiments with additional, potent siRNAs (see below).

Next, we used a BrdU incorporation assay to analyze the effect of stable BCL9L down-regulation on the proliferation of pancreatic cancer cells. In Panc-1 cells depletion of BCL9L expression led to a significant reduction of cell proliferation rates by 40.4% ± 15.8 in comparison to control cells (Figure 2C).

Besides proliferation the ability of pancreatic cancer cells to migrate as well as to invade through a layer of extracellular matrix directly correlates with their metastatic potential. Hence we performed wound-healing and transwell invasion assays to study the effect of BCL9L downregulation on these metastatic phenotypes. The results revealed that the migratory and invasive properties of Panc-1 cells were severely impaired upon knockdown of BCL9L (20 ± 4% and 50 ± 10%, respectively; Figure 2D–2E).

### BCL9L knockdown does not affect transcriptional activity of β-catenin in pancreatic cancer cells

**Figure 3:**
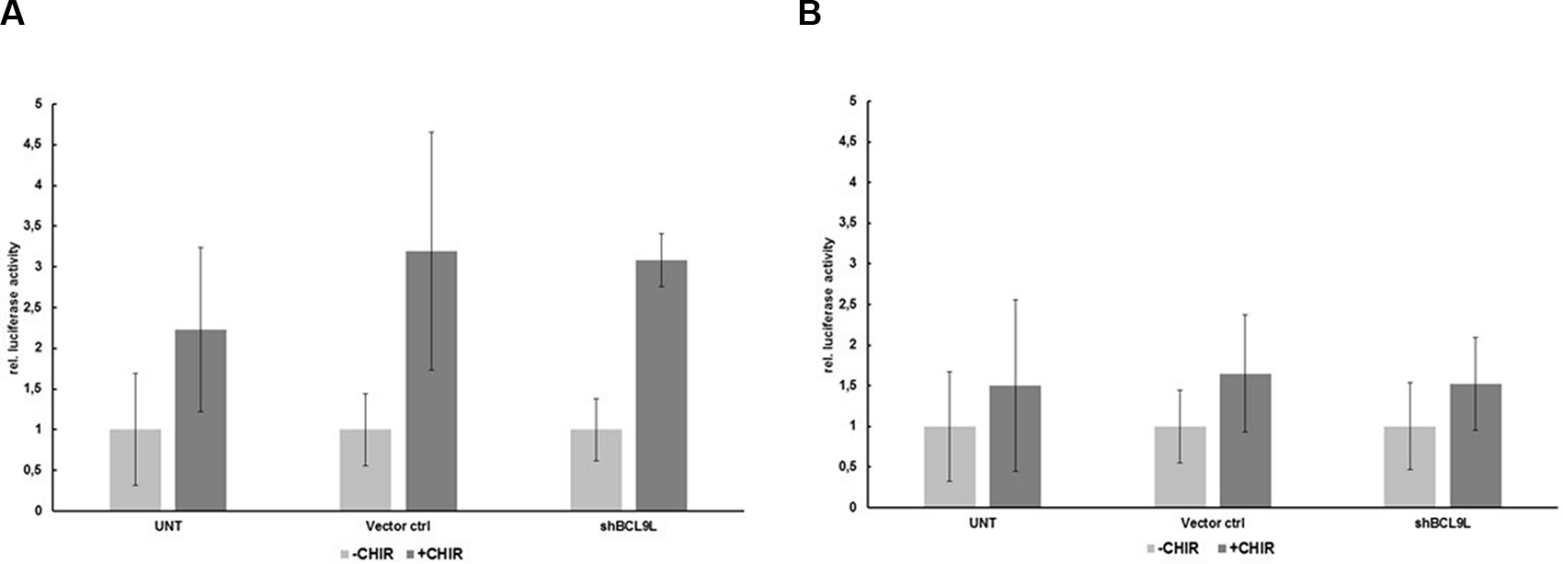
BCL9L knockdown does not affect transcriptional activity of β-catenin in pancreatic cancer cells Luciferase activity of reporter constructs containing wild-type (TOPFLASH; **A**) or mutated (FOPFLASH, negative control; **B**) TCF/LEF binding site repeats, respectively, in Panc-1 BCL9L-knockdown and control cells. Following transient transfection with luciferase constructs, cells were treated overnight with 10 μM CHIR inhibitor or left untreated and subjected to analysis of luciferase activity. Data represent mean ± s.e.m. of three independent experiments.

Previous studies described BCL9L as an important co-activator of WNT signaling and its importance for β-catenin-/TCF-mediated transcription in colon cancer cells [22, 29, 30]. We employed a TOPFLASH/FOPFLASH luciferase assay to determine to what extent BCL9L is important for β-catenin-/TCF-mediated transcription in pancreatic cancer cells. Hence, Panc-1 cells stably expressing BCL9L shRNA were co-transfected with respective luciferase reporter and control plasmids. Subsequently, these cells were treated with 10 μM CHIR 99021 GSK3β inhibitor [31] to increase the amount of cytosolic β-catenin or left untreated. Following cell lysis, β-catenin-dependent activation of the TOPFLASH luciferase reporter was analyzed (Figure 3A) and compared to a non-functional FOPFLASH control construct (Figure 3B). Our results show no significant difference of TOPFLASH reporter activity in BCL9L knockdown compared to control cells (Figure 3A), indicating that BCL9L seems to be dispensable for activation of β-catenin-dependent transcription in pancreatic cancer cell lines. In line with these results, qRT-PCR analyses for WNT target genes (incl. CyclinD1, c-Myc) did not reveal differences in their expression between control and BCL9L knockdown cells (data not shown).

### Loss of BCL9L up-regulates E-cadherin expression in pancreatic cancer cell lines and induces translocation of β-catenin to adherens junctions at the plasma membrane

The differential subcellular localization of β-catenin is important for the cellular function and activity of this signaling protein. While plasma membrane bound β-catenin mediates cell-cell adhesion via binding to E-cadherin and α-catenin at adherens junctions, freely diffusible β-catenin in the cytoplasm is involved in the transmission of the WNT signal. Under WNT off conditions non-membrane bound β-catenin is rapidly degraded [32, 33].

**Figure 4:**
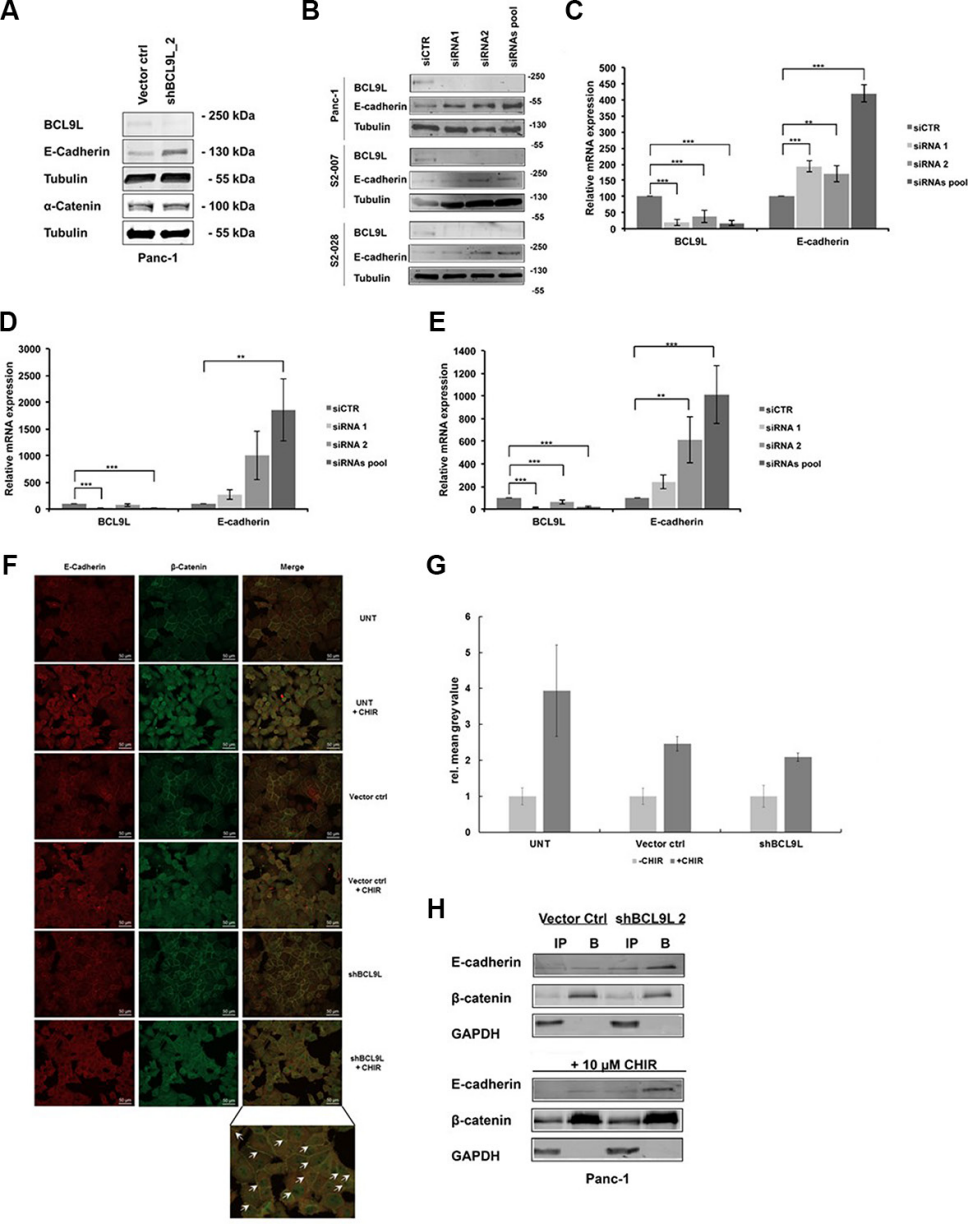
Knockdown of BCL9L stimulates E-cadherin expression and plasma-membrane retention in complex with β-catenin E-cadherin, α-catenin, BCL9L and α-tubulin (loading control) protein levels were determined in stably shRNA-transduced Panc-1 cells (**A**) as well as S2-007, S2-028 and Panc-1 cells transiently transfected with siRNA (**B**). Knockdown of BCL9L by both shRNA (shBCL9L) and siRNA (siRNA1, siRNA2, siRNA pool) induced an up-regulation of E-cadherin protein in all cell lines compared to controls (Vector control, siCTR). Shown are representative images of 3 western blot experiments. qRT-PCR analyses of BCL9L and E-cadherin mRNA expression levels in Panc-1 (**C**), S2-007 (**D**), and S2-028 (**E**) cells transfected with control siRNA or BCL9L-targeting siRNA showed a significant up-regulation of E-cadherin expression in response to knockdown of BCL9L. Data represent mean ± s.e.m. of three independent experiments. **p ≤ 0.01, ***p ≤ 0.001 (Student's *t-test*). (**F**) Untransduced (UNT), Vector control and BCL9L-shRNA transduced Panc-1 cells were treated or not with 10 μM CHIR and subjected to immunofluorescence co-staining of E-cadherin (left panel; visualized by Alexa 555-tagged secondary antibody) and β-catenin (middle panel; visualized by Alexa 488-tagged secondary antibody), respectively. Irrespective of CHIR treatment BCL9L-knockdown cells show increased retention of E-cadherin and β-catenin staining at the plasma membrane compared to controls (see arrows, bottom right panel). (**G**) Quantification of CHIR-induced nuclear translocation of β-Catenin in samples from (F). Data shown represent mean and standard deviation of mean grey values from nuclear ROIs of each 20 cells quantified as described in Materials and Methods. (**H**) β-catenin was immunoprecipitated from total cell lysates of Panc-1 BCL9L-knockdown and control cells treated or not with CHIR inhibitor. Western Blot analysis of β-catenin, E-cadherin and GAPDH (control) protein levels in input (IP) and bound (B) fractions of the immunoprecipitation revealed increased binding of E-cadherin to β-catenin protein in presence and absence of CHIR in BCL9L knockdown cells compared to controls. Shown is a representative image of 2 experiments.

Since our data did not show a significant impairment of β-catenin-dependent gene transcription upon loss of BCL9L expression (Figure 3), we wondered whether BCL9L knockdown might influence the subcellular distribution of β-catenin in pancreatic cancer cells. As the BCL9L binding site has been shown to overlap with the E-cadherin binding site of β-catenin [34], we hypothesized that loss of BCL9L expression might affect the integrity of the β-catenin nuclear complex resulting in increased levels of β-catenin bound to E-cadherin at adherens junctions at the plasma membrane. For this aim, we first analyzed E-cadherin protein and mRNA expression in Panc-1 BCL9L knockdown and control cells. Indeed, our data show a significant up-regulation of E-cadherin protein expression following BCL9L knockdown (Figure 4A). Importantly, protein levels of α-catenin, another important binding partner of β-catenin at adherens junctions remained unchanged upon BCL9L knockdown (Figure 4A). In line with results obtained from shRNA-transduced Panc-1 cells, transient transfection of BCL9L siRNA induced a complete abrogation of BCL9L protein (Figure 4B) and mRNA expression which was accompanied by a concomitant up-regulation of E-cadherin expression in all cell lines tested (Figure 4B–4E). These results strongly suggest that expression of E-cadherin is up-regulated upon knockdown of BCL9L in pancreatic cancer cells.

Next, we sought to determine whether an up-regulation of E-cadherin as a result of BCL9L knockdown would lead to subcellular re-organization of β-catenin. For this, we performed immunofluorescence stainings for β-catenin and E-cadherin in Panc-1 control and BCL9L knockdown cells (Figure 4F). Image analysis of untreated and BCL9L knockdown cells revealed co-localization of both proteins at the plasma membrane implicating the presence of β-catenin at adherens junctions of pancreatic cancer cells. In support of our hypothesis, our data further show an increase of β-catenin protein levels at the plasma membrane of BCL9L knockdown cells as compared to control cells. Treatment of cells with the GSK3β inhibitor CHIR clearly activated nuclear translocation of β-catenin with no detectable difference between control and BCL9L knockdown cells (Figure 4F and quantification of nuclear β-catenin signal in Figure 4G). This observation is in line with the results obtained from TOPFLASH/FOPFLASH luciferase assays (Figure 3), in which BCL9L knockdown did not affect transcriptional activity of β-Catenin. At the same time, even after CHIR treatment we observed increased retention of β-catenin and E-Cadherin at the plasma membrane in BCL9L knockdown compared control cells (Figure 4F). To further validate this result we performed co-immunoprecipitation experiments. Panc-1 cells comprising wild-type levels or after depletion of BCL9L were either left untreated or incubated with CHIR. Subsequently cells were lysed and the soluble protein fractions were subjected to immunoprecipitation using an immobilized β-catenin-specific nanobody [35]. Input and bound fractions were subjected to Western Blotting and membranes were probed for β-catenin and E-cadherin. In support of our immunofluorescence data the results show that upon knockdown of BCL9L E-cadherin is strongly co-precipitated with β-catenin irrespective of CHIR inhibitor treatment. In contrast, only a minor fraction of β-catenin seems to interact with E-cadherin in cells comprising wild-type levels of BCL9L (Figure 4H). Together, these data consistently suggest that loss of BCL9L induces a subcellular re-distribution of β-catenin with increased retention at the plasma membrane of pancreatic cancer cells.

### BCL9L depletion counteracts TGF-β-induced EMT *in vitro*

**Figure 5:**
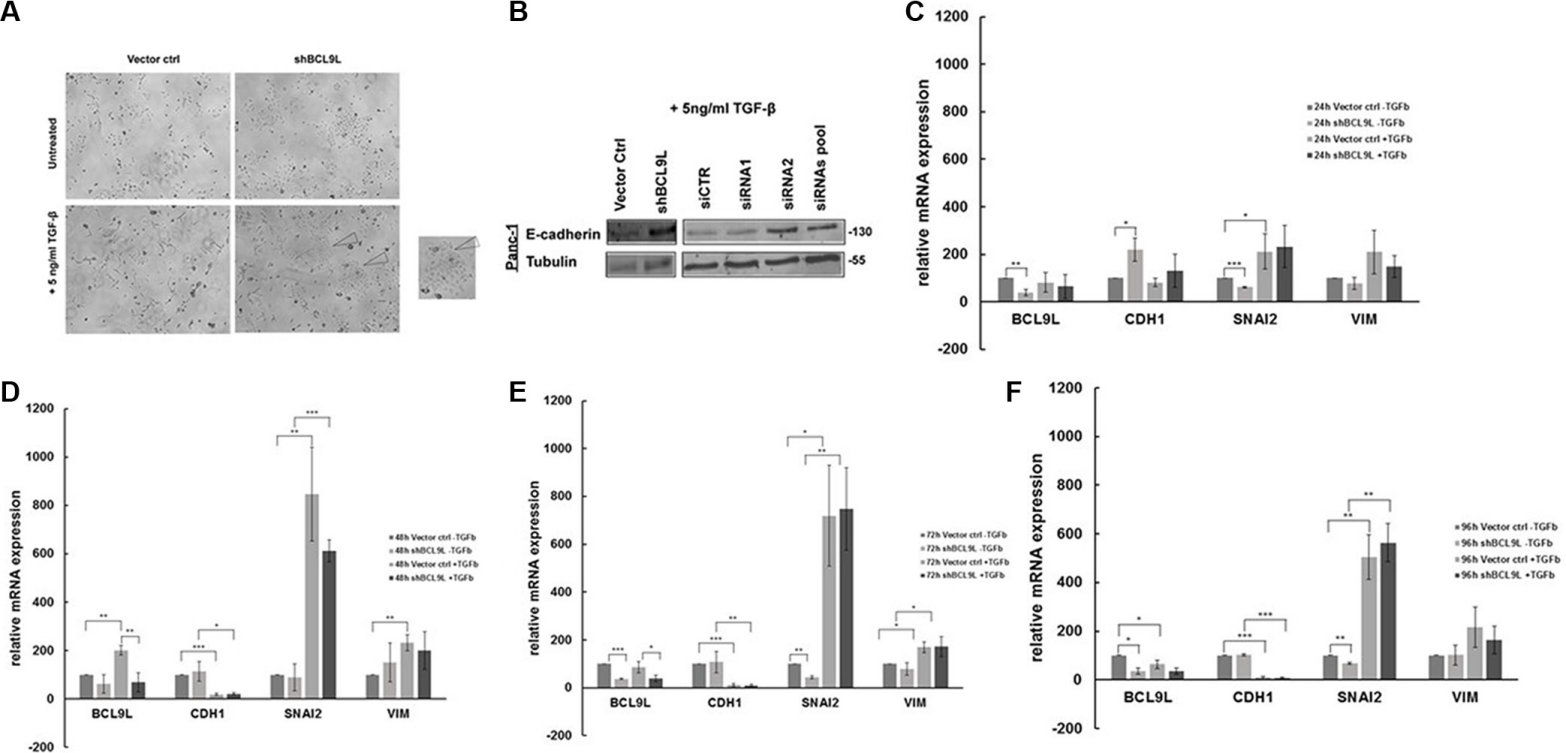
RNAi-mediated inhibition of BCL9L expression counteracts epithelial-mesenchymal transition in pancreatic cancer cells treated with TGF-β (**A**) Panc-1 cells stably transduced with control vector or BCL9L shRNA (shBCL9L) were treated with 5 ng/ml TGF-β or left untreated for 72 h and subsequently visualized using a phase-contrast microscope. Control cells responded to TGF-β treatment by adopting a mesenchymal, spindle-like phenotype whereas BCL9L-knockdown cells largely retained the cobblestone-like epithelial morphology. (**B**) Western Blot analysis of E-cadherin and α-tubulin (loading control) protein levels in Panc-1 cells treated with 5 ng/ml TGF-β for 72 h. shRNA- and siRNA-mediated knockdown of BCL9L (shBCL9L, siRNA1, siRNA2, siRNA pool) induced an upregulation of E-cadherin expression in comparison to controls (Vector ctrl, siCTR). Shown are representative images of 2 experiments. mRNA expression levels of epithelial (CDH1) and mesenchymal (SNAI2, VIM) genes were quantified in BCL9L-knockdown and control Panc-1 cells treated or not with TGF-β for 24 h (**C**), 48 h (**D**), 72 h (**E**) and 96 h (**F**), respectively. Data shown represent mean and standard deviation of 3 independent experiments. *p ≤ 0.05, **p ≤ 0.01, ***p ≤ 0.001 (Student's *t-test*).

Loss of E-cadherin expression and reduced cell-cell adhesion are hallmarks of the epithelial to mesenchymal transition (EMT) process, a fundamental event in tumor metastasis [9]. The results obtained so far implicate that BCL9L is involved in the regulation of EMT in pancreatic cancer cells, as its knockdown induced up-regulation of the epithelial marker protein E-cadherin accompanied by increased cell-cell adhesion. Thus, we sought to investigate whether up-regulation of BCL9L expression might fuel EMT and consequently the metastatic spread of pancreatic cancer cells. For this, we induced the EMT process in Panc-1 control and BCL9L knockdown cells by treatment with TGF-β for 72 h. Images obtained with phase-contrast microscopy showed the induction of a mesenchymal, i.e. elongated morphology with loss of defined cell-cell contacts in control cells, whereas BCL9L knockdown cells conserved a predominant epithelial phenotype with typical cobblestone-like morphology (Figure 5A). In congruence with these results, western blot analysis of whole cell lysates showed an up regulation of E-cadherin in BCL9L knockdown compared to control cells. This phenotype was also detectable after EMT induction by TGF-β treatment (Figure 5B). Next, we performed qPCR analyses from control and BCL9L knockdown cells treated with TGF-β for up to 96 h (Figure 5C–5F) to unravel differences in EMT response kinetics upon depletion of BCL9L. In line with our previous data, BCL9L knockdown induced a significant up-regulation of E-Cadherin expression in parallel to a significant down-regulation of the mesenchymal gene SNAI2. Furthermore and in accordance with the literature [9] TGF-β treatment of control Panc-1 cells induced a time-dependent, significant downregulation of E-Cadherin expression and a concomitant induction of mesenchymal gene expression (SNAI2, VIM) in pancreatic cancer cells. Albeit not significantly, this EMT response was diminished in BCL9L knockdown cells with initially increased levels of E-Cadherin and reduced SNAI2 expression (Figure 5C–5D). Interestingly, we observed a significant up-regulation of BCL9L in response to TGF-β treatment for 48 h in parallel to an up-regulation of mesenchymal SNAI2 gene expression. Taken together, reduced transcriptional repression of E-Cadherin via Snail -family transcription in BCL9L knockdown cells would explain our observation of increased E-Cadherin protein levels after TGF-β treatment as effects on mRNA expression are expected to precede changes in protein levels.

From these results we conclude that BCL9L expression is decisive for the ability of pancreatic cancer cells to undergo EMT *in vitro*.

### BCL9L knockdown inhibits pancreatic cancer growth and liver metastasis *in vivo*

**Figure 6:**
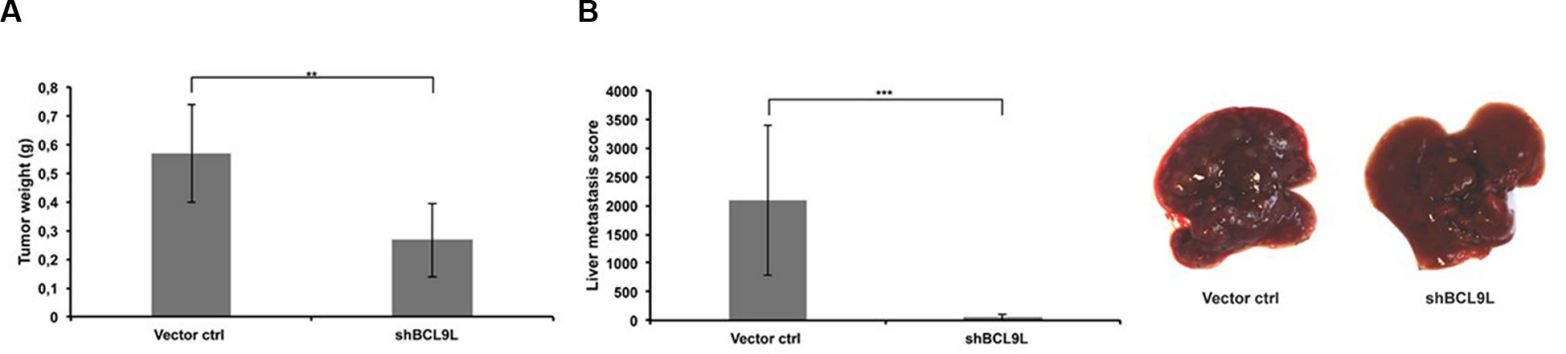
BCL9L knockdown inhibits growth and abrogates liver metastasis of pancreatic cancer cells *in vivo* (**A**) Orthotopic xenografts of Panc-1 control and BCL9L knockdown cells were established in NMRI:nu/nu mice (6 mice per group) as described in the Methods section. 5 weeks after cell implantation tumor weights were determined and revealed a significantly reduced tumor mass in mice xenografted with BCL9L knockdown cells compared to controls. (**B**) Panc-1 BCL9L knockdown and control cells were injected into the tail vein of NMRI:nu/nu mice. 5 weeks after injection, liver metastases were scored. BCL9L-knockdown significantly impaired implantation of Panc-1 cells to the liver. Right panel: representative photographs of livers from BCL9L-knockdown xenograft mice (right) illustrate the reduction in number and size of tumor nodules compared to controls (left). Data represent mean ± s.e.m. of experimental groups each representing 6 animals. **p ≤ 0.01, ***p ≤ 0.001 (Student's *t-test*).

Our findings underline the importance of BCL9L for growth and invasion of pancreatic cancer cells and their ability to undergo and complete the EMT process *in vitro*. At this point, we sought to determine whether BCL9L is implicated in growth and metastasis of pancreatic cancer cells *in vivo*. For this aim, orthotopic xenograft mouse models of Panc-1 control and BCL9L knockdown cells were generated. After 36 days, the mice were sacrificed and tumor weights were measured. We observed a significant reduction of tumor weight by 50% in mice harboring BCL9L knockdown xenografts compared to controls (Figure 6A). This result is in agreement with our *in vitro* data obtained from proliferation assays (see Figure 2C).

In a further experiment, we assessed the impact of BCL9L depletion on Panc-1 cell implantation and subsequent growth in the liver, i.e. on their ability to form metastases *in vivo*. For this, Panc-1 control and BCL9L knockdown cells were injected into the tail vein of immunocompromised mice. Liver scoring after 36 days revealed the generation of clearly visible, large metastases in control animals. Strikingly, an almost complete abrogation of metastasis formation was observed for BCL9L knockdown cells (Figure 6B). Our observation of liver metastases from BCL9L knockdown cells, albeit in reduced numbers and size, indicates that implantation is strongly diminished but not fully prevented by BCL9L depletion.

In support of the data obtained *in vitro*, these results point towards a central importance of BCL9L for growth and liver metastasis of pancreatic cancer cells *in vivo*.

## DISCUSSION

In the present study we identify a novel molecular function of BCL9L as a critical modulator of invasion and metastasis of pancreatic cancer cells *in vitro* and *in vivo*. Our data show that BCL9L knockdown correlates with a concomitant up-regulation of E-cadherin protein leading to its prolonged interaction with β-catenin at adherens junctions of the plasma membrane. In consequence, the response of BCL9L knockdown cells to EMT induction by TGF-β was delayed and accompanied by an abrogated proliferative, migratory and invasive capacity.

The human BCL9 gene was originally identified as a target of translocation t(1;14)(q21;q32) in B cell malignancies leading to its increased expression [36]. Subsequent studies identified the segment polarity gene Legless as the Drosophila orthologue [37] and BCL9L [21] as an additional member of the vertebrate BCL9 family. The amino acid sequence of BCL9L protein shows 35% overall identity to human BCL9. 90% sequence identity was observed in seven domains including the binding sites of β-catenin and Pygopus [25, 38].

To our knowledge, our study is the first one investigating the expression and functional relevance of BCL9L in human pancreatic cancer. Our data reveal significant over-expression of BCL9L in pancreatic carcinoma tissue samples compared to non-cancer controls. Moreover, the intensity of BCL9L staining in PDAC tissue samples significantly correlated with differentiation grade of the tumor.

Analysis of a panel of pancreatic cancer cell lines shows a consistently higher expression of BCL9L in comparison to non-cancer cell lines and compared to its homologue BCL9 (data not shown). In support of our findings, mining of datasets from previous expression array analyses through the Oncomine^®^ database (www.oncomine.org, May 2015, Thermo Fisher Scientific, Ann Arbor, MI) indicates a highly significant overexpression (*p* < 0.01) of BCL9L in pancreatic carcinoma compared to normal pancreas tissue derived from 52 [39] and 78 patients [40], respectively. In both studies BCL9L ranked among the top 4% of target genes overexpressed in pancreatic carcinoma.

With regard to its functional importance in human malignancies it has been shown earlier that BCL9L overexpression increases cell migration in MDCK cells [25] and siRNA-induced BCL9L knockdown inhibited proliferation of MCF7 breast cancer cells [26]. Similarly, RNAi-mediated knockdown of the BCL9L homologue BCL9 in colon cancer and multiple myeloma cell lines led to inhibition of cell proliferation, migration and invasion [41]. In line with these results, our data show a significant reduction of pancreatic cancer cell proliferation, as measured by BrdU incorporation, as well as impaired migration and transwell invasion of pancreatic cancer cells upon knockdown of BCL9L.

As a member of the BCL9/Legless protein family, BCL9L has been shown to function in conjunction with Pygopus proteins as a nuclear co-activator of canonical WNT/β-catenin signaling [38, 42]. Due to the presence of highly conserved regions involved in the interaction of BCL9 family members with β-catenin and Pygopus it was initially proposed that BCL9, Legless as well as BCL9L have redundant functions as co-activating adaptor molecules bridging β-catenin to Pygopus, which then recruits additional components of the β-catenin transcriptional complex [43]. However, subsequent studies challenged this view by showing that the function of BCL9 family members in vertebrates is only in part dependent on their interaction with Pygopus [24, 26, 44]. The results of our study did neither reveal a significant impairment of transcriptional activity nor of nuclear translocation ability of β-catenin in pancreatic cancer cells following knockdown of BCL9L. Moreover, we did not observe significant effects of BCL9L knockdown on the expression of BCL9 (data not shown). Both BCL9 and BCL9L are known to bind to β-catenin and trigger the interaction of β-catenin with the TCF/LEF-transcriptional complex [43]. Hence, knockdown of BCL9L expression would in consequence not necessarily result in reduced β-catenin-dependent TCF/LEF-activation but might be compensated by BCL9 expression. This would, however, not explain the clear functional consequences we observed on WNT/β-catenin-dependent cellular processes including reduced migration, proliferation and invasion of pancreatic cancer cells upon BCL9L knockdown. Thus, we hypothesized that this phenotype might be due to effects on the junctional pool of β-catenin which is known to be associated with α-catenin and E-cadherin at the plasma membrane [45]. Intriguingly, substantial overlap between the E-cadherin and BCL9/BCL9L binding site of β-catenin has been described [34]. Our data confirm that knockdown of BCL9L leads to an up-regulation of E-cadherin expression with concomitant down-regulation of SNAI2 gene expression in pancreatic cancer cells. In support of our hypothesis further results revealed that this pool of E-cadherin is increasingly associated with β-catenin at the plasma membrane. Additionally, significant amounts of β-catenin were retained at the plasma membrane and remained in complex with E-cadherin even in the presence of a GSK3β inhibitor which induces the enrichment of freely diffusible β-catenin in the cytosol and consequently its nuclear translocation. Previous work in MDCK cells has shown nuclear translocation of β-catenin following over-expression of BCL9L [25] reasoning an importance of this protein for the modulation of adhesive and transcriptional activity of β-catenin. However, the relevance of BCL9L expression for the integrity of adherens junctions in cancer cells has not been investigated before. Generally, cadherins are thought to dampen WNT pathway activation by sequestering β-catenin to the plasma membrane [18]. Recently, however, the requirement of E-cadherin for WNT signaling [46] as well as the transduction of pro-proliferative signals via an E-cadherin-dependent Yap1/β-catenin cascade [47] has been demonstrated. In support of a similar situation in pancreatic cancer cells, we did not observe an inhibition of the β-catenin-dependent transcriptional response upon knockdown of BCL9L despite of increased binding of β-catenin to E-cadherin. High levels of E-cadherin at the plasma membrane are generally considered as an “epithelial” phenotype characterized by polarized and basal membrane–anchored cells with cobblestone-like morphology [48]. During the process of epithelial-mesenchymal-transition (EMT) external stimuli as for example EGF and TGF-β have been shown to induce a phenotypic transformation of cells accompanied by down-regulation of epithelial signature proteins (incl. E-cadherin) and induction of a mesenchymal phenotype with increased expression of N-cadherin and transcription factors of the Snail and Zeb protein family [49]. The fact that BCL9L knockdown induced a sustained upregulation of E-cadherin expression led us to further investigate a possible modulation of the EMT process by loss of BCL9L. Indeed, our data show that down-regulation of BCL9L inhibits induction of EMT by TGF-β in pancreatic cancer cells. BCL9L knockdown cells preserved an epithelial phenotype and increased protein expression levels of E-cadherin even in the presence of TGF-β. Results from further kinetic gene expression analyses revealed that the transcriptional response of pancreatic cancer cells towards TGF-β with down-regulation of E-Cadherin and up-regulation of mesenchymal genes is delayed upon BCL9L depletion. Importantly, Slug expression is reduced in BCL9L knockdown cells already in the absence of TGF-β. Snail-family genes are well-known repressors of E-Cadherin expression, induced in response of TGF-β signaling [13, 14]. A reduction in their expression upon BCL9L knockdown would explain the observed upregulation of E-Cadherin in pancreatic cancer cells. Surprisingly, our data also show an initial up-regulation of BCL9L expression in response to TGF-β treatment in control cells concomitant with an up-regulation of mesenchymal gene (Slug, Vimentin) expression. This up-regulation is followed by the reduction of BCL9L and mesenchymal gene expression during long-term treatment with TGF-β. Intensive interaction of TGF-β and WNT signaling pathways on multiple levels has been described [11, 50]. It is tempting to speculate that transcriptional regulation of BCL9L expression in response to TGF-β represents another point of crosstalk in pancreatic cancer.

In the past years, accumulating evidence suggested that the activation of the EMT process is of central importance for metastasis of human malignancies with TGF-β being the major inducer of EMT in pancreatic cancer [51]. Recently, it has been demonstrated that induction of TGF-β release from Kupffer cells by PDAC-derived exosomes is fundamental to the formation of the pre-metastatic niche in the liver [52]. In support of our *in vitro* data, results from *in vivo* xenograft experiments showed reduced tumor growth of BCL9L knockdown cells. Importantly, the capacity of BCL9L knockdown cells to form metastases in the liver was almost completely abrogated, which further suggests a central importance of BCL9L during TGF-β induced EMT in pancreatic cancer cells. To our knowledge this is the first study demonstrating an upregulation of E-cadherin expression and its increased association with β-catenin in response to BCL9L knockdown.

In summary, we identify BCL9L as a novel regulator of TGF-β-induced EMT in pancreatic cancer. In light of recently described selective inhibitors with high specificity for β-catenin/BCL9 interactions [53] our results provide evidence that direct targeting of this complex might be a valuable approach to effectively antagonize invasion and metastasis of this malignancy.

## MATERIALS AND METHODS

### Cell culture and TGF-β treatment

Panc-1, S2-007 and S2-028 cells were kindly provided by Malte Buchholz (University of Marburg, Germany) and cultured in DMEM high glucose (Life Technologies) supplemented with 10% FCS, 1% penicillin-streptomycin and 1% glutamine. MiaPaca-2 cells were obtained from ATCC and cultured in DMEM flemented with 10% FCS, 1% penicillin-streptomycin, 1% glutamine and 2.5% horse serum. For TGF-β-treatment, cells were serum-starved for 24 h and treated with TGF-β (Peprotech) at a final concentration of 5 ng/ml for indicated time points in respective medium containing 0.5% FCS.

### Lentivirus production

Panc-1 cells were stably transduced with lentiviral pLKO.1- and TRC2-pLKO-based shRNA constructs (Sigma-Aldrich), containing a puromycin resistance cassette. Lentiviral particles were generated in HEK 293FT cells (ATCC) by using polyethylenimine (PEI) reagent. The virus-containing supernatant was collected and pooled to infect the respective target cell lines. Following infection, 2 μg/ml of puromycin was added for selection of transduced cells. Stable cell lines were maintained in growth media containing 1 μg/ml puromycin.

### RNA interference and luciferase reporter assays

For RNA interference experiments Panc-1, S2-007 and S-028 were transiently transfected with siRNA control, two distinct BCL9L or β-catenin siRNAs and a BCL9L or β-catenin siRNA pool (Thermo Fisher Scientific), respectively, at a final concentration of 20 nM using Lipofectamine RNAiMAX (Life Technologies). For luciferase reporter assays, cells were co-transfected with TOPFLASH- or FOPFLASH-luciferase reporters together with Renilla control using TransIT-LT1 reagent (Mirus Bio LLC). 24 h after transfection cells were treated over night with 10 μM CHIR 99021 (Tocris) and subjected to a Dual-Glo Luciferase Assay (Promega). Obtained firefly luciferase values were normalized to respective Renilla controls.

### Quantitative reverse-transcription PCR (qRT-PCR)

Total RNA was isolated from respective cell lines using the RNeasy mini kit (Qiagen) and reverse-transcribed using M-MuLV Reverse Transcriptase (New England Biolabs). qRT-PCR was performed using gene-specific qPCR assays comprising ZEN double-quenched probes (IDT) on a 7500 Fast Real-Time PCR System (Applied Biosystems). Gene expression was calculated relative to GAPDH as internal control. Tissue samples were homogenized in liquid nitrogen using a mortar and pestle and RNA extracted using the RNeasy mini Kit (Qiagen) following the manufacturer's protocol. 1 μg total RNA was used for first-strand cDNA synthesis using the Omniscript RT Kit (Qiagen). qRT-PCR was performed using SYBR Green MasterMix (Applied Biosystems) on a 7500 Fast Realtime PCR system (Applied Biosystems).

### Western blotting, immunoprecipitation and immunofluorescence staining

For western blot analysis, cells were lysed using ice-cold RIPA buffer (150 mM NaCl, 1% Triton X-100, 0.5% Na-dexycolate, 0.1% SDS, and 50 mM Tris pH 8.0). Lysate protein concentration was quantified using BCA protein assay reagent (Thermo Scientific). 30 μg of total protein from each sample was resolved by SDS-PAGE and transferred to nitrocellulose membranes (GE Healthcare) using semi-dry transfer (Peqlab). Membranes were blocked in 5% milk powder, incubated overnight with primary antibody diluted in 5% BSA, washed with 0.05% TBS-T and incubated with an Alexa Fluor 647-conjugated secondary antibody (Molecular Probes). Bands were visualized using a Typhoon laser scanner (GE Healthcare). For immunofluorescence experiments cells were grown in 96-well μClear plates (Greiner), treated as indicated, washed three times with D-PBS and fixed by addition of 4% paraformaldehyde solution in D-PBS for 15 min at room temperature. Next, cells were permeablilised using 0.5% Triton-X-100 in D-PBS for 5 min and washed three times with D-PBS. To prevent non-specific antigen binding, cells were incubated with 5% BSA/D-PBS for 30 minutes followed by extensive washing with D-PBS. Subsequently, samples were incubated with respective primary antibodies diluted in BSA/D-PBS at 4°C overnight. Antibody-signals were visualized using Alexa−488 and −546 conjugated secondary antibodies, respectively, diluted in D-PBS. Samples were imaged on a Cell Observer spinning-disc confocal microscope (Zeiss). For image analysis the Fiji platform [54, 55] was used. Regions of interest (ROIs) covering nuclear localizations were defined and signal intensities (mean grey values) measured in *n* = 20 independent cells from *n* = 3 replicate experiments (arbitrary units).

For co-immunoprecipitation experiments 1 × 10^6^ Panc-1 cells stably expressing a shRNA vector control (vector ctrl) or a BCL9L specific shRNA (shBCL9L) were either left untreated or incubated with CHIR-99021 (Tocris Bioscience) dissolved in H_2_O for 24 h. Cells were washed and harvested in phosphate buffered saline (PBS), snap-frozen in liquid nitrogen and stored at −20°C. Cell pellets were homogenized in 200 μl lysis buffer (10 mM Tris/Cl pH7.5, 150 mM NaCl, 0.5% NP40, 1 μg DNaseI, 2 mM MgCl_2_, 2 mM PMSF, 1× phosSTOP phosphatase inhibitor (Roche), 1× protease inhibitor mix M (Serva) by repeated pipetting for 40 min on ice. After a centrifugation step (10 min at 18.000 × g, 4°C) the protein concentration of each lysate was determined using Coomassie Plus according to manufacturer's protocol (Thermo Fisher Scientific) and the protein solutions were adjusted with dilution buffer (10 mM Tris/Cl pH 7.5, 150 mM NaCl, 2 mM PMSF) to equal concentrations. 2% of the lysate were added to SDS-containing sample buffer (referred to as input, IP). For co-immunoprecipitation 50 μl of the β-catenin-specific nanobody immobilized on a sepharose matrix [35] were added and incubated for 12 h on an end-over-end rotor at 4°C. The bead pellet was washed two times in 0.5 ml dilution buffer. After the last washing step the beads were transferred to a new cup, resuspended in 2× SDS-containing sample buffer and boiled for 10 min at 95°C. Samples (0.5% input, 10% bound) were analyzed by SDS-PAGE followed by western blotting. Immunoblots were probed with indicated anti-E-cadherin, anti-β-catenin and anti-GAPDH antibodies for normalization of the input fractions and as negative control to detect unspecific binding to the sepharose matrix.

### Antibodies

The following antibodies were used in this study: BCL9L (sheep polyclonal, # AF4967, R&D Systems; HPA 049370, Atlas Antibodies), α-tubulin (mouse monoclonal, B-5-1-2, #T5168, Sigma Aldrich), β-catenin (rabbit polyclonal, H102, #sc-7199, Santa Cruz), E-cadherin (goat polyclonal, #AF648, R&D Systems), α-catenin (mouse monoclonal, 6D202, #C2069-44H, USBiological).

### Proliferation, migration and transwell invasion assays

BrdU incorporation was measured using the chemiluminescent cell proliferation ELISA (Roche) according to manufacturer's instructions. Cells were seeded in 96-well plates at 5 × 10^3^ cells/well and were cultured for 4 days. Cell migration was assessed using a wound healing assay. Briefly, cells were grown in 6 well-plates until confluence. A wound was generated by scraping with a 200 μl pipet-tip. Subsequently, phase-contrast images were acquired immediately after scratching and then every 24 h until the wound was closed in the control treated samples. The wound size was calculated using T-scratch software [56]. Cell invasion was analyzed using a 24-multiwell insert system (Corning). 8 μm pore inserts were coated with 0.4 mg/ml of matrigel (BD). Cells were seeded into the upper chamber at a concentration of 5 × 10^4^ cells/well in 350 μl serum-free medium. The lower chamber was filled with 750 μl DMEM supplemented with 10% FCS. Following incubation at 8% CO_2_, 37°C for 24 h matrigel and cells on top of the transwell insert were removed with a cotton-tipped swab. Cells which invaded onto the lower side of the membrane were fixed with methanol, stained with crystal violet solution and counted. The invasion index was calculated by dividing the percentage of crystal-violet positive BCL9L knockdown cells by the percentage of respective control cells.

### Immunohistochemistry

Tissue microarray construction and immunohistochemistry have been performed as described before [57].

For the BCL9L immunostaining a rabbit polyclonal antibody (HPA 049370, Atlas Antibodies) has been used. Prior to the incubation with the primary antibody (1:100, overnight) heat induced antigen retrieval in citrate buffer (pH 6.5) was performed. Kidney tissue was used as positive control. The intensity of the staining reactions was scored as mild, moderate or strong (score 1, 2 or 3, respectively). The proportion of the positive cells in ducts and tumor areas was estimated in percent and divided into scores (< 10% −1, 10–50% −2, 51–80% −3, > 80% −4). The final score was determined as a product of the intensity of the staining and the proportion of positive cells (minimum 0, maximum 12) as described previously [58]. Scores of BCL9L expression were analyzed by Mann-Whitney and Kruskal-Wallis non parametric tests, respectively. Results were calculated using GraphPad Prism 4.0 software (La Jolla, CA, USA).

### *In vivo* tumorigenicity

All mice experiments were carried out by the EPO GmbH, Berlin-Buch. The animal experiments were performed according to the German Animal Protection Law and with approval from the responsible authorities. The *in vivo* procedures were consistent and in compliance with the UKCCCR guidelines. Briefly, for analysis of tumorigenic growth of indicated Panc-1 cell lines, 1 × 10^6^ cells were intravenously injected into the tail of NMRI:nu/nu mice (6 mice per cell line). In a parallel experimental set-up the cells were injected orthotopically. For this, 5 × 10^5^ cells were mixed with matrigel to a final volume of 40 μl and carefully injected into the pancreatic tail of narcotized NMRI:nu/nu mice (Anesthesia: hypnomidate, i.p.). For both experimental settings, cells were rinsed through a 70 μm cell strainer before implantation. NMRI:nu/nu mice had an age of 8 weeks at time of transplantation. The health of mice was examined daily and body weight was taken twice a week. On day of necropsy mice were sacrificed by cervical dislocation and inspected for gross organ changes. General criteria for termination of metastasis experiments: poor general condition, moribundity, body weight loss or abnormal behavior. The primary tumor (i.panc.) and tumoral affected organs were dissected, weighted and snap-frozen for further analyses. The metastatic burden in mice organs was evaluated using a metastasis score (based on number and size of metastatic nodules).

## References

[1] Seufferlein T, Bachet JB, Van Cutsem E, Rougier P (2012). Pancreatic adenocarcinoma: ESMO-ESDO Clinical Practice Guidelines for diagnosis, treatment and follow-up. Ann Oncol.

[2] Bond-Smith G, Banga N, Hammond TM, Imber CJ (2012). Pancreatic adenocarcinoma. BMJ.

[3] Becker AE, Hernandez YG, Frucht H, Lucas AL (2014). Pancreatic ductal adenocarcinoma: risk factors, screening, and early detection. World J Gastroenterol.

[4] Malvezzi M, Bertuccio P, Rosso T, Rota M, Levi F, La Vecchia C, Negri E (2015). European cancer mortality predictions for the year 2015: does lung cancer have the highest death rate in EU women?. Ann Oncol.

[5] Muniraj T, Jamidar PA, Aslanian HR (2013). Pancreatic cancer: a comprehensive review and update. Dis Mon.

[6] Stathis A, Moore MJ (2010). Advanced pancreatic carcinoma: current treatment and future challenges. Nat Rev Clin Oncol.

[7] Rhim AD, Mirek ET, Aiello NM, Maitra A, Bailey JM, McAllister F, Reichert M, Beatty GL, Rustgi AK, Vonderheide RH, Leach SD, Stanger BZ (2012). EMT and dissemination precede pancreatic tumor formation. Cell.

[8] Oberstein PE, Olive KP (2013). Pancreatic cancer: why is it so hard to treat?. Therap Adv Gastroenterol.

[9] Ye X, Weinberg RA (2015). Epithelial-Mesenchymal Plasticity: A Central Regulator of Cancer Progression. Trends Cell Biol.

[10] Zhou B, Liu Y, Kahn M, Ann DK, Han A, Wang H, Nguyen C, Flodby P, Zhong Q, Krishnaveni MS, Liebler JM, Minoo P, Crandall ED (2012). Interactions between beta-catenin and transforming growth factor-beta signaling pathways mediate epithelial-mesenchymal transition and are dependent on the transcriptional co-activator cAMP-response element-binding protein (CREB)-binding protein (CBP). J Biol Chem.

[11] Fuxe J, Vincent T, Garcia de Herreros A (2010). Transcriptional crosstalk between TGF-beta and stem cell pathways in tumor cell invasion: role of EMT promoting Smad complexes. Cell Cycle.

[12] Kim MK, Maeng YI, Sung WJ, Oh HK, Park JB, Yoon GS, Cho CH, Park KK (2013). The differential expression of TGF-beta1, ILK and wnt signaling inducing epithelial to mesenchymal transition in human renal fibrogenesis: an immunohistochemical study. Int J Clin Exp Pathol.

[13] Wang Y, Shi J, Chai K, Ying X, Zhou BP (2013). The Role of Snail in EMT, Tumorigenesis. Curr Cancer Drug Targets.

[14] Zheng H, Kang Y (2014). Multilayer control of the EMT master regulators. Oncogene.

[15] Liu Z, Habener JF (2010). Wnt signaling in pancreatic islets. Adv Exp Med Biol.

[16] Jones S, Zhang X, Parsons DW, Lin JC, Leary RJ, Angenendt P, Mankoo P, Carter H, Kamiyama H, Jimeno A, Hong SM, Fu B, Lin MT (2008). Core signaling pathways in human pancreatic cancers revealed by global genomic analyses. Science.

[17] Waddell N, Pajic M, Patch AM, Chang DK, Kassahn KS, Bailey P, Johns AL, Miller D, Nones K, Quek K, Quinn MC, Robertson AJ, Fadlullah MZ (2015). Whole genomes redefine the mutational landscape of pancreatic cancer. Nature.

[18] Orsulic S, Huber O, Aberle H, Arnold S, Kemler R (1999). E-cadherin binding prevents beta-catenin nuclear localization and beta-catenin/LEF-1-mediated transactivation. J Cell Sci.

[19] Aberle H, Butz S, Stappert J, Weissig H, Kemler R, Hoschuetzky H (1994). Assembly of the cadherin-catenin complex *in vitro* with recombinant proteins. J Cell Sci.

[20] Ring A, Kim YM, Kahn M (2014). Wnt/catenin signaling in adult stem cell physiology and disease. Stem Cell Rev.

[21] Katoh M, Katoh M (2003). Identification and characterization of human BCL9L gene and mouse Bcl9l gene in silico. Int J Mol Med.

[22] Adachi S, Jigami T, Yasui T, Nakano T, Ohwada S, Omori Y, Sugano S, Ohkawara B, Shibuya H, Nakamura T, Akiyama T (2004). Role of a BCL9-related beta-catenin-binding protein, B9L, in tumorigenesis induced by aberrant activation of Wnt signaling. Cancer Res.

[23] de la Roche M, Worm J, Bienz M (2008). The function of BCL9 in Wnt/beta-catenin signaling and colorectal cancer cells. BMC Cancer.

[24] Brembeck FH, Wiese M, Zatula N, Grigoryan T, Dai Y, Fritzmann J, Birchmeier W (2011). BCL9–2 promotes early stages of intestinal tumor progression. Gastroenterology.

[25] Brembeck FH, Schwarz-Romond T, Bakkers J, Wilhelm S, Hammerschmidt M, Birchmeier W (2004). Essential role of BCL9–2 in the switch between beta-catenin's adhesive and transcriptional functions. Genes Dev.

[26] Zatula N, Wiese M, Bunzendahl J, Birchmeier W, Perske C, Bleckmann A, Brembeck FH (2014). The BCL9–2 proto-oncogene governs estrogen receptor alpha expression in breast tumorigenesis. Oncotarget.

[27] Deer EL, Gonzalez-Hernandez J, Coursen JD, Shea JE, Ngatia J, Scaife CL, Firpo MA, Mulvihill SJ (2010). Phenotype and genotype of pancreatic cancer cell lines. Pancreas.

[28] Taniguchi S, Iwamura T, Katsuki T (1992). Correlation between spontaneous metastatic potential and type I collagenolytic activity in a human pancreatic cancer cell line (SUIT-2) and sublines. Clin Exp Metastasis.

[29] de la Roche M, Ibrahim AE, Mieszczanek J, Bienz M (2014). LEF1 and B9L shield beta-catenin from inactivation by Axin, desensitizing colorectal cancer cells to tankyrase inhibitors. Cancer Res.

[30] Sakamoto I, Ohwada S, Toya H, Togo N, Kashiwabara K, Oyama T, Nakajima T, Ito H, Adachi S, Jigami T, Akiyama T (2007). Up-regulation of a BCL9-related beta-catenin-binding protein, B9L, in different stages of sporadic colorectal adenoma. Cancer Sci.

[31] Ring DB, Johnson KW, Henriksen EJ, Nuss JM, Goff D, Kinnick TR, Ma ST, Reeder JW, Samuels I, Slabiak T, Wagman AS, Hammond ME, Harrison SD (2003). Selective glycogen synthase kinase 3 inhibitors potentiate insulin activation of glucose transport and utilization *in vitro* and *in vivo*. Diabetes.

[32] Hagen T, Sethi JK, Foxwell N, Vidal-Puig A (2004). Signalling activity of beta-catenin targeted to different subcellular compartments. Biochem J.

[33] Krieghoff E, Behrens J, Mayr B (2006). Nucleo-cytoplasmic distribution of beta-catenin is regulated by retention. J Cell Sci.

[34] Sampietro J, Dahlberg CL, Cho US, Hinds TR, Kimelman D, Xu W (2006). Crystal structure of a beta-catenin/BCL9/Tcf4 complex. Mol Cell.

[35] Traenkle B, Emele F, Anton R, Poetz O, Haeussler RS, Maier J, Kaiser PD, Scholz AM, Nueske S, Buchfellner A, Romer T, Rothbauer U (2015). Monitoring interactions and dynamics of endogenous beta-catenin with intracellular nanobodies in living cells. Mol Cell Proteomics.

[36] Willis TG, Zalcberg IR, Coignet LJ, Wlodarska I, Stul M, Jadayel DM, Bastard C, Treleaven JG, Catovsky D, Silva ML, Dyer MJ (1998). Molecular cloning of translocation t(1;14)(q21;q32) defines a novel gene (BCL9) at chromosome 1q21. Blood.

[37] Townsley FM, Cliffe A, Bienz M (2004). Pygopus and Legless target Armadillo/beta-catenin to the nucleus to enable its transcriptional co-activator function. Nat Cell Biol.

[38] Kramps T, Peter O, Brunner E, Nellen D, Froesch B, Chatterjee S, Murone M, Zullig S, Basler K (2002). Wnt/wingless signaling requires BCL9/legless-mediated recruitment of pygopus to the nuclear beta-catenin-TCF complex. Cell.

[39] Pei H, Li L, Fridley BL, Jenkins GD, Kalari KR, Lingle W, Petersen G, Lou Z, Wang L (2009). FKBP51 affects cancer cell response to chemotherapy by negatively regulating Akt. Cancer Cell.

[40] Badea L, Herlea V, Dima SO, Dumitrascu T, Popescu I (2008). Combined gene expression analysis of whole-tissue and microdissected pancreatic ductal adenocarcinoma identifies genes specifically overexpressed in tumor epithelia. Hepatogastroenterology.

[41] Mani M, Carrasco DE, Zhang Y, Takada K, Gatt ME, Dutta-Simmons J, Ikeda H, Diaz-Griffero F, Pena-Cruz V, Bertagnolli M, Myeroff LL, Markowitz SD, Anderson KC (2009). BCL9 promotes tumor progression by conferring enhanced proliferative, metastatic, and angiogenic properties to cancer cells. Cancer Res.

[42] Thompson BJ (2004). A complex of Armadillo, Legless, and Pygopus coactivates dTCF to activate wingless target genes. Curr Biol.

[43] Hoffmans R, Basler K (2007). BCL9–2 binds Arm/beta-catenin in a Tyr142-independent manner and requires Pygopus for its function in Wg/Wnt signaling. Mech Dev.

[44] Cantu C, Zimmerli D, Hausmann G, Valenta T, Moor A, Aguet M, Basler K (2014). Pax6-dependent, but beta-catenin-independent, function of Bcl9 proteins in mouse lens development. Genes Dev.

[45] McCrea PD, Maher MT, Gottardi CJ (2015). Nuclear signaling from cadherin adhesion complexes. Curr Top Dev Biol.

[46] Howard S, Deroo T, Fujita Y, Itasaki N (2011). A positive role of cadherin in Wnt/beta-catenin signalling during epithelial-mesenchymal transition. PLoS One.

[47] Benham-Pyle BW, Pruitt BL, Nelson WJ (2015). Cell adhesion. Mechanical strain induces E-cadherin-dependent Yap1 and beta-catenin activation to drive cell cycle entry. Science.

[48] Gheldof A, Berx G (2013). Cadherins and epithelial-to-mesenchymal transition. Prog Mol Biol Transl Sci.

[49] Thiery JP, Acloque H, Huang RY, Nieto MA (2009). Epithelial-mesenchymal transitions in development and disease. Cell.

[50] Zhang J, Tian XJ, Xing J (2016). Signal Transduction Pathways of EMT Induced by TGF-beta, SHH, and WNT, Their Crosstalks. J Clin Med.

[51] Jiang JH, Liu C, Cheng H, Lu Y, Qin Y, Xu YF, Xu J, Long J, Liu L, Ni QX, Yu XJ (2015). Epithelial-mesenchymal transition in pancreatic cancer: Is it a clinically significant factor?. Biochim Biophys Acta.

[52] Costa-Silva B, Aiello NM, Ocean AJ, Singh S, Zhang H, Thakur BK, Becker A, Hoshino A, Mark MT, Molina H, Xiang J, Zhang T, Theilen TM (2015). Pancreatic cancer exosomes initiate pre-metastatic niche formation in the liver. Nat Cell Biol.

[53] Zhang M, Wisniewski JA, Ji H (2015). AlphaScreen selectivity assay for beta-catenin/B-cell lymphoma 9 inhibitors. Anal Biochem.

[54] Schindelin J, Rueden CT, Hiner MC, Eliceiri KW (2015). The ImageJ ecosystem: An open platform for biomedical image analysis. Mol Reprod Dev.

[55] Schindelin J, Arganda-Carreras I, Frise E, Kaynig V, Longair M, Pietzsch T, Preibisch S, Rueden C, Saalfeld S, Schmid B, Tinevez JY, White DJ, Hartenstein V (2012). Fiji: an open-source platform for biological-image analysis. Nat Methods.

[56] Geback T, Schulz MM, Koumoutsakos P, Detmar M (2009). TScratch: a novel and simple software tool for automated analysis of monolayer wound healing assays. Biotechniques.

[57] Palagani V, Bozko P, El Khatib M, Belahmer H, Giese N, Sipos B, Malek NP, Plentz RR (2014). Combined inhibition of Notch and JAK/STAT is superior to monotherapies and impairs pancreatic cancer progression. Carcinogenesis.

[58] Kaistha BP, Lorenz H, Schmidt H, Sipos B, Pawlak M, Gierke B, Kreider R, Lankat-Buttgereit B, Sauer M, Fiedler L, Krattenmacher A, Geisel B, Kraus JM (2016). PLAC8 Localizes to the Inner Plasma Membrane of Pancreatic Cancer Cells and Regulates Cell Growth and Disease Progression through Critical Cell-Cycle Regulatory Pathways. Cancer Res.

